# Core Outcome Set development for LEPtospirosis trials (COS-LEP): a study protocol to develop a core outcome set for the evaluation of clinical therapeutic interventions for human leptospirosis

**DOI:** 10.1186/s13063-024-08713-6

**Published:** 2025-01-06

**Authors:** Nathaniel Lee, Chris Smith, Robin Bailey, Koya Ariyoshi, Sarah Smith, Nick Black, Tansy Edwards

**Affiliations:** 1 Faculty of Epidemiology and Population Health, London School of Hygiene & Tropical Medicine, London, UK; 2 School of Tropical Medicine and Global Health, Nagasaki University, Nagasaki, Japan; 3 Faculty of Infectious and Tropical Diseases, London School of Hygiene & Tropical Medicine, London, UK; 4 Department of Clinical Medicine, Institute of Tropical Medicine (NEKKEN), Nagasaki University, Nagasaki, Japan; 5 Department of Health Services Research & Policy, London School of Hygiene & Tropical Medicine, London, UK; 6 MRC International Statistics and Epidemiology Group, Faculty of Epidemiology and Population Health, London, School of Hygiene & Tropical Medicine , London, UK

**Keywords:** Leptospirosis, Core outcome set, Clinical trial, Systematic review, Qualitative interview, Delphi survey

## Abstract

**Background:**

Leptospirosis is a zoonotic bacterial infection occurring worldwide. It is of particular public health concern due to its global distribution, epidemic potential and high mortality without appropriate treatment. The method for the management of leptospirosis, particularly in severe disease, is clouded by methodological inconsistency and a lack of standardized outcome measures.

The study this protocol details aims to develop a core outcome set (COS) for leptospirosis research. A COS is a set of outcomes with international consensus as a minimum for reporting in future studies focusing on leptospirosis. Establishing a COS will contribute to harmonizing Leptospirosis treatment research and will be instrumental in constructing a high-quality evidence base to feed into a planned future rigorous international clinical trial on leptospirosis.

**Methods:**

The COS-LEP study will employ a COS development methodology standardized by the COMET initiative framework. This includes (1) a systematic review of available quantitative and qualitative literature reporting therapeutic response and safety outcomes and measures; (2) focused interviews with healthcare professional and people treated for leptospirosis exploring outcomes of interests using qualitative methodology; (3) narrowing the choice of outcomes by international consensus using a Delphi survey process; and (4) undertaking a hybrid consensus meeting with key stakeholders to build the final COS.

**Discussion:**

This protocol describes the method to develop the first core outcome set for use in human leptospirosis studies. This will not only be a key feature in the design of a future definitive randomized controlled trial, but also provide a structure for clinicians and researchers collecting treatment cohort data in the various settings where leptospirosis is a public health issue.

**Supplementary Information:**

The online version contains supplementary material available at 10.1186/s13063-024-08713-6.

## Introduction

Leptospirosis is a zoonotic bacterial infection occurring worldwide caused by *Leptospira interrogans*. There are an estimated 1.03 million cases and 58,900 deaths due to Leptospirosis worldwide per year [1] Although sporadically reported in high-income countries, low- and middle-income countries bear a high burden of cases—particularly in the South East Asian, Pacific, and Central and South American geographical areas. More frequent climate catastrophes, poor infrastructure and social deprivation in these areas often precipitate outbreaks and epidemics [1].

The diagnosis of leptospirosis is difficult. Clinical manifestations vary from mild to severe disease requiring hospitalization and organ-supported care. Early manifestations are of an acute febrile illness (mimicking the presentation of various systemic infections in the tropics) with 10% of infected patients developing severe disease, including manifestations of hepato-renal failure and pulmonary haemorrhage requiring intensive hospital care [2].

The evidence for the management of leptospirosis particularly in severe disease is limited by methodological inconsistency and a lack of established outcome measures to characterize disease and evaluate treatments. A 2012 Cochrane review of antimicrobial treatment included seven randomized trials spanning 1988–2007. The outcome measures used varied from mortality, length of hospital stay, urinary culture clearance, resolution of biochemical changes, and defervescence (i.e. resolution of fever). Despite four trials including individuals with severe disease, no definitions of severity were provided. Duration of symptoms varied at presentation in each study. The review concluded there was insufficient evidence to advocate for, or against, the use of antimicrobials due to poor methodological quality or underpowered studies [3].

Similarly, when considering immunosuppressive therapies for severe leptospirosis disease, the last systematic review conducted in 2014 concluded there was insufficient data to establish efficacy [4]. Outcomes measures varied for these studies and included death, duration of ventilation, and duration of bleeding.

The establishment of core outcome sets (COS) to define outcomes to be used in a clinical trial is increasingly important to improve relevance of trials to health service users and policy makers [5]. This is particularly vital for research activities involving neglected diseases, as the availability of fewer trials means that robust methods must be established to ensure reliable comparability between available trial results in a consistent manner [6]. To date there have been no COS established specifically for the clinical management of leptospirosis. Standardization of measures would add consistency and lead to harmonization of leptospirosis therapeutic trials, thereby reducing waste in production and reporting of research. Furthermore, there are currently no established primary efficacy or safety outcomes for a definitive clinical trial of leptospirosis treatments, a significant contributing factor to current inconclusive systematic reviews regarding treatment and which represents a neglected research area.

The aim of this study protocol is to describe a study framework that will define the minimum agreed upon set of outcomes for any clinical therapeutic trial in human leptospirosis.

## Methods

### Scope

To develop the COS-LEP protocol guidance set out by the Core Outcome Measures in Effectiveness Trials (COMET) initiative, and further specified by the Core Outcome Set-Standardised Protocol Items (COS-STAP) statement, were followed [7, 8]. These provide standardised frameworks for the development of a COS. The COMET database (available at https://www.comet-initiative.org/Studies) was searched for any pre-existing COS protocols for leptospirosis. When it was confirmed that there were none, this protocol was then registered (https://www.cometinitiative.org/Studies/Details/2536) with a project start date of June 2023 and an end date of October 2025.

The population that will be targeted in this COS study are any individuals working in healthcare who regularly look after patients diagnosed with leptospirosis disease, researchers and policymakers involved in leptospirosis research, and individuals or groups representing individuals who are at risk of or who have been diagnosed with leptospirosis disease.

**Fig. 1:**
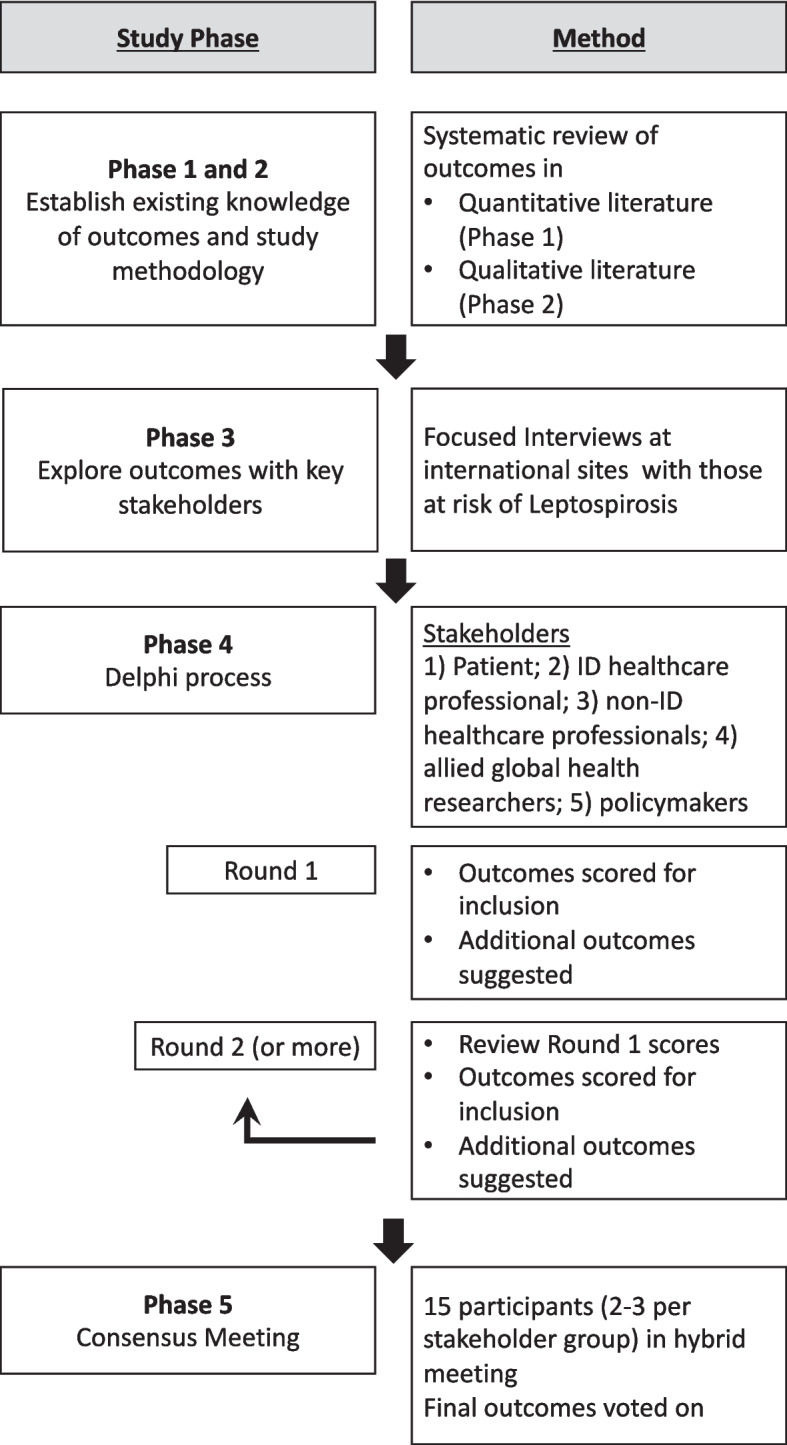
Summary of COS-LEP study

The project will be carried out in five distinct phases, summarized in Fig. 1. The combined Phase 1 and 2 will consist of undertaking a systematic review of the literature for reported outcomes from quantitative and qualitative studies on the management of leptospirosis. In Phase 3, focused interviews to explore further outcomes of interest will be undertaken with healthcare providers and people at risk of leptospirosis infection. Phase 4 will consist of a multi-round Delphi survey process to begin constructing consensus around the outcome set. And Phase 5 will finalize the process with a consensus meeting.

The reporting recommendations of the Standard Protocol Items: Recommendations for Interventional Trial statement (SPIRIT) were used to guide the design of this protocol.[9] The SPIRIT checklist is provided as a supplementary document (Supplementary 1).

### Phase 1 and 2

Phase 1 and 2 will establish existing knowledge about outcomes and methodology used in previous quantitative and qualitative studies looking at the treatment of human leptospirosis. To explore outcomes, a systematic review of outcomes from previously published quantitative and qualitative studies will be undertaken. The protocol for this systematic review has already been registered with the PROSPERO system in detail (PROSPERO ID CRD42023397461). In addition, systematic Cochrane Library reviews will be completed exploring methodology of all known clinical trials for human leptospirosis treatment, and determine efficacy and safety outcomes reported to date for evaluations of therapeutic interventions.[10–12] The output from all of these initiatives will provide the basis for options to put forward at later agreement stages.

### Study selection

The following databases will be included in the search: the Cochrane Database of Systematic Reviews, Cochrane Central Register of Controlled Trials, MEDLINE, EMBASE, LILACS, Web of Science Core Collection, Clinicaltrials.gov, and OpenSIGLE. The latter will be particularly important for the identification of grey literature sources. In each identified study, forward and backward reference searching will be used to identify further relevant publications.

Any study focusing on human leptospirosis will be included, across all age groups in a global distribution. Non peer-reviewed studies, most likely grey literature sources, may be considered. We will assess studies in batches chronologically by descending years in 5-year frames, until we achieve saturation in outcome types.

Full manuscripts will be prioritized, but abstract-only publications may be considered particularly if the full publication is unable to be obtained from the publisher or manuscript author. The search will not be initially limited by language, but studies may be excluded depending on which languages reviewing authors are able to speak. We will report the results of our literature search in line with the Preferred Reporting Items for Systematic reviews and Meta-Analyses Statement for Reporting Literature Searches in Systematic Reviews (PRISMA-S) checklist [13].

### Data extraction

Studies will be screened using the Covidence systematic review software (https://www.covidence.org/). Title and abstract review, followed by full text review, will be performed by two reviewers. A third reviewer will be consulted if there is a disagreement between the first two reviewers that cannot be resolved through discussion.

Data will be collected on the following;

1. Study design and sample size
2. Study population—demographics, location
3. Intervention
4. Outcomes
5. Outcome measurements instruments and/or definitions provided by the authors for each outcome
6. Study conclusions

### Quality assessment

The primary author will assess the quality of included studies in accordance to the QualSyst tool [14]. Risk of bias in outcome reporting will be assessed according to the Outcome Reporting Bias in Trials (ORBIT) study classification system, and the ultimate goal will be to provide context to outcome measures rather than a basis for study exclusion [15].

### Data analysis

Data describing study design, population, treatments, and outcome measures will be summarized with descriptive statistics. Data on outcome will be extracted verbatim. These will then be grouped based on terminology into group names, and then further grouped into domains [8]. With respect to outcomes, as names and domains are dependent on the outcomes identified, these will not be defined a priori. A narrative synthesis of the included studies will be performed focused on the types of data collected defined above. The identified groupings will be mapped to the outcome taxonomy framework proposed by the COMET Initiative [8, 16]. Subgroup analysis will be considered if there are defined populations targeted by a particular outcome.

### Phase 2 specific considerations

COMET handbook guidelines recommend the systematic review and identification of studies that specifically explore outcomes important to health service users [8]. These may either be in the form of previously published Patient-Reported Outcome Measures (PROMs) or studies employing qualitative methodology.

It is not expected that a significant number of qualitative studies exploring treatment outcomes or studies establishing PROMs for leptospirosis have been previously published. If identified, a similar study selection and appraisal process as described in Phase 1 will be employed, focused on studies with a qualitative or mixed-methods design (where qualitative data collection and analysis methods were employed). With regard to data extraction, previously published methodology for extracting and analysing outcomes from studies reporting PROMs [17] and qualitative literature [18–21] will be used. Concepts and themes from excerpts will be categorized and tabulated to obtain frequency information. Categories will be mapped against the proposed COMET taxonomy, and if multiple domains are appropriate then two domains will be chosen as per guidance and precedence [8, 19].

### Phase 3

Central to the COS-STAP statement is the definition of stakeholder groups who will be involved in the COS development process including how individuals will be identified, and the nature of their involvement [7]. This is particularly important as it is expected that identification of PROMs through the systematic review of qualitative literature may not yield many results.

Therefore, Phase 3 will involve exploring outcomes of interest with healthcare providers and end-users directly affected by leptospirosis, to identify outcomes important to these groups, through the use of qualitative methods. This will, in addition, identify target population groups, explore acceptability of therapeutic interventions, and assess feasibility of conducting trial activities.

#### Participants recruitment and sample size

Participant identification and recruitment will be done from collaborative institutional sites in the COS-LEP network. The institutions in the network are secondary/tertiary hospitals primarily found in areas endemic for leptospirosis—the Philippines, Malaysia, Vietnam, New Caledonia, and Brazil. This may extend to other institutional sites in the same or different countries during the COS study (with appropriate local ethics agreements obtained prior to any enrolment activities).

Participants who are healthcare providers that manage patients with leptospirosis will be identified and approached for recruitment. Additionally those at risk of leptospirosis, or who have made contact with healthcare services and for whom leptospirosis is included in the differential diagnosis, will also be identified and approached for recruitment. They will be provided with a patient information leaflet, as well as a verbal description of the study and its intention. After a 24-h consideration period, they will be approached again and consented for enrolment.

Purposive sampling will be employed. Sample size calculations are a point of contention in qualitative studies, but methodological literature recommends that the reasoning behind any calculation be made transparent [22]. A report on current COS methodological guidance states that the focus of the qualitative component in COS studies is not on quantification of incidence by sampling, but on collecting rich data that will allow in-depth exploration [23]. Data collection will normally continue until a point of diminishing returns is achieved. The Core Outcome Development for Carriers Screening (CODECS) study achieved theoretical sufficiency with 15 participants across five countries [24]. The Core outcome Set for Physiotherapy trials in adults with BronchiEctasis (COS-PHyBE) study achieved a data saturation with 18 participants across four countries [25]. Both the CODECS and COS-PHyBE studies recruited participants from various countries, conducted semi-structured individual interviews, and were able to obtain sufficient data with their sample sizes to explore their participant’s perspectives in detail. The COS-LEP study will employ a similar approach, recruiting participants from international sites where collaborative institutional research is currently ongoing. Based on the sampling for the CODECS and COS-PHyBE studies, we will aim to recruit at least 6 participants from each of our participating sites. This may be revised should further sites be identified. The overall aim will be to achieve theoretical sufficiency, defined as a point when a researcher has achieved sufficient or adequate depth of understanding to build a theory [22, 26].

#### Data collection

Semi-structured interviews will be conducted. These will either be in person or remote/via teleconferencing means, the latter which now has a growing body of evidence supporting its use in qualitative methodology [27]. For those wishing to participate, but have limited fluency in English, attempts will be made to provide an interpreter subject to availability of personnel and/or funding. A very broad topic guide will be used, which will include direct exploration of outcomes and outcome measures, but the interview will be guided primarily by participants. Interviews will be audio recorded, and then anonymized and transcribed using the NVivo software (QRS International). Once all transcriptions are complete, interview recordings will be securely destroyed.

#### Data analysis

Thematic analysis will be employed to summarize transcripts [28]. Sections in the transcripts involving outcomes will be coded, subsequent codes collated into potential themes, themes revised and then defined. The generated themes will then be mapped against the outcome taxonomy proposed by the COMET Initiative, although consideration will be made to include if there is no adequate classification. Common themes in the classifications will be identified and outcome definitions that closely approximate each other will be removed.

### Phase 4

The next stage of the COS development process will obtain a broad consensus on the proposed set of core outcomes identified in Phase 1–3. The recommended methodology to accomplish this is the Delphi technique targeted at experts and other stakeholders through the use an evolving questionnaire and multiple rounds of consensus surveys [8].

#### Participant recruitment and sample size

The stakeholders of interest for participation in surveys are patients (those who experienced leptospirosis disease), infectious diseases healthcare professionals (including medical microbiologist), non-infectious diseases healthcare professionals (including nephrology, pulmonology, intensive care, family medicine or other specialties), allied global health researchers (including epidemiologists, trialists, statisticians, and One Health practitioners), and policymakers (both governmental and non-governmental). As many stakeholders as possible in a worldwide geographic spread will be included. Guidance on total number of stakeholders to be included is not based on statistical power but a pragmatic choice [8]. We will seek to include sufficient participants per group to achieve good representation, but which will still allow for a consensus within group to be achieved.

Stakeholders will be approached for recruitment using several communication avenues both in-person and virtual. Participation in surveys will be advertised to patients by direct means (at collaborative sites and those identified during Phase 3), dissemination through hospital communications at collaborative sites, through a project-specific website, and through social media. Researchers active in human leptospirosis will be identified from studies collated in Phase 1–2 of the COS process. Healthcare professionals of any specialities will be identified by direct means at collaborative sites and through various professional networks of research teams. Policymakers will be identified through recommendation, existing national/international leptospirosis guidance, and from committee membership or involvement on various international leptospirosis societies. All participants will be requested to disseminate information through their various networks in order to increase uptake.

Potential participants expressing interesting in taking part in the survey will be directed to a study page provided by the Qualtrics platform that will provide study information in lay terms with full definition of medical terminology if used. Basic information such as demographics and suitability to participate will be assessed if potential participants wish to proceed. If these are met, then they will be able to proceed to the Delphi survey, with consent being gained electronically before undertaking the questionnaire.

#### Data collection

The COMET Initiative has offered guidance that at least two survey rounds take place in order to incorporate feedback [8]. The minimum number of rounds for this study will be two but will be extended if further consensus is required. The survey will be built using the Qualtrics survey software (https://www.qualtrics.com) with a focus on lay language minimizing medical jargon (or with definitions included). We will attempt to engage with patients/members of the public identified from previous Phases of the COS-LEP study to develop and trial this lay terminology. Translations into other languages for particular groups will be explored depending on the participant groups included. Trials of the survey will be run amongst the study group and colleagues, and feedback incorporated back into the survey design.

The COMET initiative suggests the use of the 9-point Likert scale where outcomes are graded in accordance to their level of importance, which is also the recommended framework by the Grading of Recommendations Assessment, Development and Evaluation (GRADE) Working Group [8, 29]. This uses a scale between 1 and 9, with number 1–3 defined as outcome of limited importance, 4–6 outcome of importance but not critical, and 7–9 meaning critical outcome. There will be an additional grade of “Unable to score” should participants feel they do not have the level of expertise to score a certain outcome [8]. The order of outcomes to be scored might introduce a bias, and so outcomes order will be randomized as used in other COS studies [19]. Space will be provided at the point of rating each outcome and at the end of the questionnaire for participants to list additional outcomes not considered or to provide feedback on the list of outcomes. These will be reviewed between rounds by the study group and select patient representatives to determine classification within COMET taxonomy, how different the proposed outcome is from the existing listed outcome, and ultimately whether appropriate for inclusion in next round.

There is wide variability in definition of whether individual outcomes meet a consensus for inclusion or exclusion of subsequent surveys, or at the end of the study. The COMET handbook does not provide a clear framework, but suggests more inclusive criteria be used in subsequent survey rounds should items be excluded in the intervening period [8]. Relevant results from the first survey round will be presented to participants in the second round, presented graphically and by stakeholder group. This will enable participants to compare their prior scores within their stakeholder group and with other groups in the survey process. This study will adopt criteria used in previous COS studies, with less stringent criteria in earlier rounds [19, 30–32]. Common in all rounds will be (1) the definition of consensus for exclusion from next round, which is any outcome with ≥ 70% of all participants rated 1–3 (not important) for an outcome in any round; (2) any score not meeting inclusion or exclusion criteria will defined as no consensus; and (3) A participant’s score will be included for analysis if they complete 70% or more of outcomes.

- **Round 1.** Inclusion in next round/phase of study—any outcome rated 7–9 (critical outcome) by > 50% of any participant and 1–3 (limited importance) by no more than 15% of any single stakeholder group.
- **Round 2.** Inclusion in next round/phase of study—any outcome rated 7–9 (critical outcome) by > 70% of any participant, and 1–3 (limited importance) by no more than 15% of any single stakeholder group.

One further round of surveys may be implemented after careful consideration by the COS-LEP steering group under the following circumstances: if there are additional outcomes recommended by survey participants which have not been included, and if the list of outcomes remain too wide in scope as determined by the COS-LEP steering group. The Round 3 inclusion definition to take forward to a consensus meeting would remain as for Round 2 in order to maintain a stringent criteria. When deciding whether to proceed with a third round, the steering committee will consider any limitations in time or further burden on participants for entering a third Delphi round [8].

#### Addressing attrition

There is a risk of non-response by participants at all stages of the Delphi process, likely made greater by the international scope of the study. In order to decrease rate of non-responses, surveys will be kept open for 4 weeks and up to three reminder e-mails per round will be sent to participants with incomplete surveys. Details of current response rate and reminders of survey closing dates will be included in each message. If response rates remain low, consideration will be made for additional measures to increase this with consideration to time and funding. We will aim for 80% response per stakeholder group.

#### Data analysis

The distribution of outcomes for inclusion moving forward and degree of consensus will be summarized at the end of each round [8]. Means and medians for each outcome will be calculated. The change of scores between rounds will be assessed by change in percentage, reduction in spread of scores by comparing standard deviations, and reductions in interquartile range [8]. Metrics will be reported for all participants, and inter-group between stakeholders.

### Phase 5

The final phase of the COS development process will involve a consensus meeting where representatives of the various stakeholder groups can convene to discuss the results of the survey and decide on a final core set. Although we will aim for an in-person meeting, given the international scope of this study and the distribution of the stakeholder group, we may explore the possibility of conducting a hybrid approach of virtual and in-person meeting. If using a hybrid meeting approach, we will use guidance published by the COMET Initiative [33].

#### Participant recruitment and sample size

Building on the standard proposed by the CODECS study, we will aim to recruit at least 20 participants. This includes at least 4 from each stakeholder group to allow consensus to be achieved based on our revised consensus criteria. Participants will be purposively selected for inclusion in a minimum of one consensus meeting [19]. We will adjust this number depending on interest and the practicalities/logistics of attending an in-person meeting. Although any participant may be included, to provide transparency in selection, the final Delphi survey round will include a query of whether a participant is able to attend the consensus meeting. Consideration will also be made to include participants who did not respond or dropped out during the Delphi survey. We will explore the possibility of using break-out groups depending on total group size and availability of facilitators who can be trained to moderate as used in other COS development studies [34].

The final number of participants and number of meetings will be a pragmatic decision based on expressed interest in participation, feasibility of joining a meeting, and ensuring representative input from all participants.

#### Data collection

The consensus meeting will consist of an introduction of the aims of the COS-LEP study, a summary of Phase 1–4 activities and results, the discussions of the final set of outcomes, and a final vote on outcomes. The meeting will be recorded and take place over 2–3 h, but may be longer or divided into sessions depending on the number of participants, availability, and number of outcomes. Outcomes to be discussed will primarily be those that were selected in the Phase 4 Delphi survey, but outcomes which were excluded may be re-introduced and discussed again depending on participant interest.

Within each stakeholder group, > 70% votes for either direct inclusion or exclusion of outcomes will be considered consensus. If there are issues with total number of participants or a limited number of stakeholder groups attending, we may consider using a threshold of > 70% across the entire group. These criteria have been proposed by the COMET Initiative as well as other COS developers such as the Outcome Measures in Rheumatology (OMERACT) group [8, 35, 36]. Results will be presented after voting, and reported by stakeholder group as well as across the entire group. Outcomes which are excluded or which do not meet the criteria of consensus can be reviewed by participants where there is opportunity to discuss any reason to disagree with these results. Should the number of included outcomes be large, a method to classify these following previous examples such as composite or tiered outcomes will be discussed in group [36, 37]. Construction of the final outcome set will also include an agreement on outcome measurement definitions [8]. The meeting will be transcribed and analysed using thematic analysis to explore participant’s perspectives on the agreed core outcomes, barriers to implementing, and any recommendation or suggestions for proceeding [34]. The outcome of Phase 5 will be the final core outcome set.

#### Dissemination of results

The complete results of the COS study will be reported in international peer-reviewed open-access journals, international and national scientific and policymaking meetings, shared to clinical trial registries, and directly through all study group and participant networks. All study reports will follow reporting standards defined in the COS-STAR statement [5].

## Discussion

The COS-LEP study protocol describes the establishment of a COS for human interventional trials in leptospirosis disease. As highlighted earlier, the clinical management of leptospirosis is complicated by inconsistent trial methodology in published studies to date. To the authors’ knowledge, this is the first COS developed for leptospirosis trials, and can serve as a template should such standards be required in the field of animal health.

This COS will ensure that a minimum set of outcomes are included in any human leptospirosis trial. The use of these outcomes will have been agreed upon by an international group of healthcare professionals, policymakers, and patients. In this manner, the methodology of future trials can be harmonized and allow for easier and more meaningful comparison of results, particularly if undertaking meta-analysis. It will also be of benefit to the international treatment centres that manage leptospirosis cases and wish to record harmonized patient data.

## Trial status

This protocol version number is v12.0 (dated 20/11/2024). Systematic reviews quantitative and qualitative literature are in progress. Development of core outcome set is ongoing.

## Supplementary Information

Supplementary Material 1

## Data Availability

All authors will have equal access to data collected, and data will be deposited is a suitable repository at the end of the process. The protocol and individual studies will be made open-access.

## Abbreviations

COS: Core Outcome Set
COMET: Core Outcome Measures in Effectiveness Trials
COS-STAP: Core Outcome Set-Standardised Protocol Items
PROMs: Patient-Reported Outcome Measures
COS-STAR: Core Outcome Set–STAndards for Reporting
